# Spatial Assessment of Solar Radiation by Machine Learning and Deep Neural Network Models Using Data Provided by the COMS MI Geostationary Satellite: A Case Study in South Korea

**DOI:** 10.3390/s19092082

**Published:** 2019-05-05

**Authors:** Jong-Min Yeom, Seonyoung Park, Taebyeong Chae, Jin-Young Kim, Chang Suk Lee

**Affiliations:** 1 Satellite Application Division, Korea Aerospace Research Institute, 115 Gwahangno Yuseong-gu, Daejeon 34133, Korea; yeomjm@kari.re.kr (J.-M.Y.); tbchae@kari.re.kr (T.C.); 2 New and Renewable Energy Resource & Policy Center, Korea Institute of Energy Research, 152 Gajeong-ro Yuseong-gu, Daejeon 34129, Korea; jinyoung.kim@kier.re.kr; 3 Environmental Satellite Center, National Institute of Environmental Research, 42, Hwangyeong-ro, Seogu, Incheon 22689, Korea

**Keywords:** solar radiation, artificial neural network, random forest, support vector machine, deep neural network, COMS MI

## Abstract

Although data-driven methods including deep neural network (DNN) were introduced, there was not enough assessment about spatial characteristics when using limited ground observation as reference. This work aimed to interpret the feasibility of several machine learning approaches to assess the spatial distribution of solar radiation on Earth based on the Communication, Ocean, and Meteorological Satellite (COMS) Meteorological Imager (MI) geostationary satellite. Four data-driven models were selected (artificial neural network (ANN), random forest (RF), support vector regression (SVR), and DNN), to compare their accuracy and spatial estimating performance. Moreover, we used a physical model to probe the ability of data-driven methods, implementing hold-out and k-fold cross-validation approaches based on pyranometers located in South Korea. The results of analysis showed the RF had the highest accuracy in predicting performance, although the difference between RF and the second-best technique (DNN) was insignificant. Temporal variations in root mean square error (RMSE) were dependent on the number of data samples, while the physical model showed relatively less sensitivity. Nevertheless, DNN and RF showed less variability in RMSE than the others. To examine spatial estimation performance, we mapped solar radiation over South Korea for each model. The data-driven models accurately simulated the observed cloud pattern spatially, whereas the physical model failed to do because of cloud mask errors. These exhibited different spatial retrieval performances according to their own training approaches. Overall analysis showed that deeper layers of networks approaches (RF and DNN), could best simulate the challenging spatial pattern of thin clouds when using satellite multispectral data.

## 1. Introduction

The energy required to drive terrestrial processes is mostly provided by solar radiation, which is, therefore, an important factor of influence for agriculture, forest science, hydrology, and meteorology. Moreover, solar radiation not only powers photosynthesis in terrestrial ecosystems, but also drives evaporation from the surface and thus is a variable that connects land–atmosphere fluxes [1,2]. In land surface and hydrological modeling, solar radiation incident on a given surface is one of the indispensable driving factors controlling both water and heat exchanges between land and atmosphere [3]. Consequently, the reliable estimation of solar radiation is essential for the aforementioned applications. In particular, the spatial distribution of solar radiation for specific geographic areas is an important parameter to be considered in fields such as engineering, agriculture, atmospheric science, environmental science, hydrology, and renewable energy utilization [4,5,6,7,8].

The direct use of pyranometer data from ground sites is one of the simplest ways to estimate on-surface solar radiation, providing mostly accurate estimates of incoming solar radiation with high temporal resolution over established ground points [9]. However, this approach suffers from many technical and financial issues such as high costs and the need for highly skilled labor, periodical maintenance, cleaning, and calibration of solar sensors [10,11], which means that ground networks of pyranometers are typically not available in sufficiently high spatial coverage to resolve spatial patterns [12]. Although spatial data gap-filling techniques such as interpolation/extrapolation methods and kriging have been effectively used for numerous meteorological measurement sites [13,14], they are poorly suited for sparsely located ground measurement sites in complex and mountainous terrain, where the retrieval of solar radiation data to interpret complicated radiation processes is complicated by the scarcity of available information [2]. Thus, the utilization of ground pyranometers for directly estimating the spatial distribution of solar radiation is subject to fundamental limitations.

Satellite observation is considered to be an effective tool to obtain spatiotemporal data for the spatialization of solar radiation on the surface, allowing one to collect large amounts of information on the atmosphere and the underlying land surface [6,15]. Additionally, multispectral sensors installed on satellites are designed to interpret atmospheric effects such as light scattering, reflection, and absorption via Rayleigh scattering, aerosol, ozone, and water vapor, since the amount of radiation transferred through the atmosphere to the surface depends not only on the distribution of atmospheric constituents and but also on the sensitivities of their spectral response wavelengths [16,17]. In particular, satellites allow one to observe the temporal and spatial variations of cloud coverage, which is the most influential parameter affecting solar radiation on the surface [18,19]. Clouds typically exhibit high reflectance and low temperature [20] and can, therefore be indirectly characterized using visible to infrared (IR) multispectral imaging. Thus, the broad range of observation methods offered by satellites is expected to be well suited for estimating the spatial variation of solar radiation.

The results of satellite imaging can be utilized in two ways, one of which is to use the atmospheric parameters extracted from multispectral images to derive complex radiative transfer models or look up table-based models based on the physical parameterization of the radiation process [17,21,22,23]. Alternatively, one can directly combine the results of ground reference measurements with those of satellite imaging using empirical or heuristic mathematical approaches [17,19,24,25]. Herein, we focus on the comparative analysis of the spatial assessment of solar radiation using this data-driven model.

One of the earliest practical methods of calculating solar radiation on the Earth’s surface utilizes ground measurement–based empirical models [26,27] without any assumptions on the underlying data and explicit physics. Since ground measurements are considered to provide true data describing solar radiation on the surface, empirical methods (especially those utilized in statistics-based approaches) mainly used these references to determine their coefficients or weights of variables. However, the main constraint of empirical models is the limitation of generalization in remote regions, which leads to large uncertainties in the estimated spatial variation of solar radiation due to the lack of ground solar radiation records at most locations around the world [28,29].

Recently, machine learning approaches such as neural network, random forest (RF), and support vector machine (SVM) techniques have attracted increased attention and shown good performance in various fields [30,31], since in these approaches, the accuracy of data-driven dependent models is continuously improved through optimization using abundant data of ground reference sites. In particular, various machine learning approaches have been applied to estimate or predict solar radiation, not only because of their outstanding accuracy of retrieving and predicting solar radiation on the Earth’s surface [6,32] but also because these techniques are useful for making predictions in areas with non-linear system modeling and control [33,34,35,36]. Yeom et al. [19] suggested that multi-layer neural networks can be employed to correlate satellite spectral signals with the results of in situ pyranometer measurements and thus evaluate cloud-related attenuation of solar radiation, showing that that this approach furnishes more accurate results than selected physical models. However, since the determined weights of the neural network depended on the ground reference, the spatial expansion of solar radiation with the suggested method was not analyzed. Belaid and Mellit [37] applied a regression version of SVM to estimate solar radiation in an arid climate and obtained good agreements with ground measurements. Zhou et al. [2] presented an RF model–based machine learning approach for estimating daily downward solar radiation flux at the land surface over complex terrain using MODIS satellite data and showed that compared with the case of untrained ground reference sites, overall accurate results were obtained. Furthermore, a wavelet-coupled SVM model was used to predict global solar radiation with minimum temperature, maximum temperature, sunshine hours, precipitation, and evaporation from MODIS data, and showed reasonable performance [35]. 

In general, machine learning methods (also called data-driven models) are mostly based on supervised training approaches with ground pyranometer measurements, which indicates that data-driven models also depend on points of ground observations to finally determine weight values of multilayers or decision tree nodes. As in the case of empirical models, the main issue of data-driven learning models is the representativeness and generalization of spatial-area expansion during the estimation of the spatial map of solar radiation. Therefore, such accuracy may be maintained even in the process of spatialization of solar radiation when there are no ground meteorological stations available for optimization. 

With the development of high-performance computing, deep learning networks have been introduced to extract useful representations from large unlabeled datasets and have been applied for classification and regression in many fields [38,39,40]. In well-known tests for the recognition of handwritten digital text in the Mixed National Institute of Standards and Technology database, multicolumn deep convolutional neural networks achieved the best error rates (0.23%) reported so far, which signifies near-human performance [41]. The best error rate realized for deep convolutional neural networks is smaller than values of existing machine learning methods such as neural networks using randomly initialized backpropagation (1.6%) and SVM (1.4%) [38]. The high accuracy of deep learning is thought to originate from the complexity and deep structure of the involved networks [42], which means that deep architectures (more than three layers) of the employed networks are better suited for approximation of nonlinear functions than traditional shallow neural networks [43,44,45]. Thus, under identical conditions, more complicated and deeper network structures should simulate the targeted solution more effectively than other data-driven methods despite the limited number of labeled training samples. In other words, deep neural networks (DNNs) are trained with ground reference data to determine network structure and the weight of nodes in the same way as in existing machine learning approaches. However, it can be assumed that more complicated and deeper network structures are better suited for simulating the spatial distribution of solar radiation deviating from the site-specific reference points when satellite data are incorporated. Therefore, there is a need for research on the characteristics of the DNN method that is only trained at ground observation points when spatially extended using satellite data. Recently, several studies have used the deep learning method to solve a non-linear function of problems, especially for Earth environment parameters [46,47,48]; however, comparative analysis of the spatial assessment of DNNs in the solar radiation field remains untested in South Korea. 

Finally, the ability to accurately capture the spatial variation of solar radiation on the surface is important for hydrology, agriculture, and weather forecast models such as general circulation models and Weather Research and Forecasting (WRF) [3,49,50], since solar radiation is the principal energy source of Earth’s systems. Therefore, comparative analysis of the spatial assessment of solar radiation by shallow/deep neural networks is a task of high significance. Herein, we evaluate the spatial characteristics of solar radiation determined using the shallow/deep neural network approach coupled with satellite imaging for assessing spatial solar radiation over South Korea. In addition, a physical model is also applied to estimate solar radiation using satellite imaging as comparative approaches with a selected data-driven model due to its high generalization performance [6].

## 2. Study Area and Data Collection

### 2.1. Study Area Characteristics

**Figure 1:**
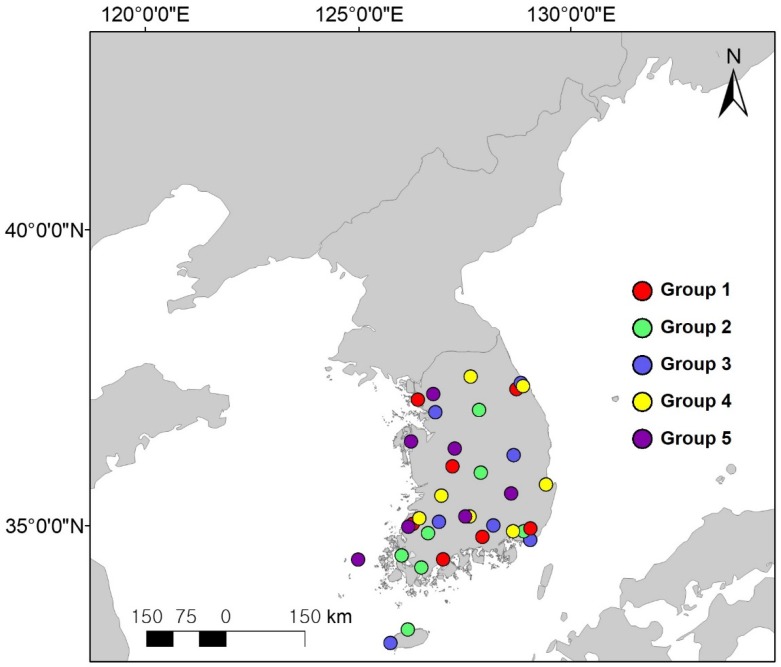
Study area, including ground station locations used for estimating solar radiation. Groups employed for five-fold cross-validation are indicated by colored circles.

The study area for the spatial assessment of solar radiation in South Korea is shown in Figure 1. The Korean Peninsula features a temperate monsoon climate that gives way to a cold continental climate (similar to that of northern China) in the northern part, whereas the southern part has a marine climate similar to that of southern Japan. Thus, as a whole, the study area is characterized by a seasonal monsoon climate. 

In this study, 35 ground pyranometers of the CM21 type produced by Kipp and Zonen were used to provide reference data for training machine learning or validating model performance. The locations of ground stations established in South Korea are shown in Figure 1; these meteorological sites were established by the Korea Meteorological Administration (KMA), and all KMA pyranometers were maintained according to World Meteorological Organization (WMO) criteria (Guide to Meteorological Instrument and Methods of Observation WMO-No. 8) to measure incoming solar radiation on the surface with high accuracy. The time range of the pyranometer observations was from April 2011 to December 2017, depending on satellite data.

### 2.2. COMS MI Satellite Data

Herein, the Communication, Ocean, and Meteorological Satellite (COMS) Meteorological Imager (MI) geostationary satellite developed by the Korea Aerospace Research Institute (KARI) was mainly used to estimate the spatiotemporal distribution of incoming solar radiation on surface. Notably, COMS MI is the first South Korean meteorological satellite that has been operated to observe various weather phenomena and thus aid weather forecasting. The location of this satellite (36,000 km altitude, 128.2° E) allows it to cover the whole Asia-Pacific area with 1-km (visible) and 4-km (infrared, IR) spatial resolution.

In this study, we used the complete L1b data set, which was acquired using completed radiative and geometric calibration and consisted of one visible (reflectance, dimensionless) and four IR (brightness temperature, kelvin) channels. The detailed characteristics of COMS MI used to estimate solar radiation in the region are listed in Table 1. The above satellite was launched on 27 June 2010, tested in orbit, and officially distributed from observed data in April 2011. Therefore, we used the L1b data of COMS MI acquired between April 2011 and December 2017. COMS MI observed the study area more than 90 times per day, and we used on-time observation data acquired during daytime.

### 2.3. Input Parameter Structure for Spatial Solar Radiation

Identical datasets were used for retrieving solar insolation by five approaches to avoid the influence of dataset difference. The dataset employed contained 14 variables that could be categorized into three classes: time (UTC, Julian day and year), data pertaining to all COMS MI bands, and geometric relationships between the sun and the surface (solar zenith angles, solar azimuth angles, viewing zenith angles, viewing azimuth angles, longitude, and latitude). To estimate solar radiation under clear-sky conditions, it is essential to consider the effects of atmospheric components and the geometric relationships between solar radiation, surface, and sensor. All bands of COMS MI were used to account for the disturbance effects due to atmospheric components. Solar radiation under cloud coverage conditions was retrieved by considering the clear-sky condition and the attenuating effects of clouds. To detect and consider clouds, we employed IR1/2 and visible bands. Originally, COMS/MI provides L1b data in a Global Earth Observation System (GEOS) projection, which shows Earth as a geostationary satellite would see it. This projection has the advantage of allowing one to see a full disk image at a glance, whereas it is not appropriate to analyze data in a high-latitude area due to its spatial distortion. Thus, we converted the GEOS projection into the World Geodetic System 1984 (WGS84) geographic projection to more easily detect the spatial distribution of solar radiation over the study area. Since solar radiation on the surface is also affected by the change of season and time because of the related variation of the incident solar angle, we accounted for these changes by tagging data with time information such as Julian day and Universal Time Coordinated (UTC).

## 3. Methods

### 3.1. Physical Model for Solar Radiation

In this study, we selected the Kawamura physical model as the base [51] and improved its cloud factor by considering the visible reflectance of clouds and the solar zenith angle instead of using brightness temperature, since cloud pass depth is more sensitive to the amount of irradiance attenuation than brightness temperature [52]. Satellite-based physical solar radiation models were used for two reasons. First, they were employed as comparative models to evaluate the generalization capability of data-driven models, since they are independent to ground measurements by explicating the physical processing of incoming radiation and its atmospheric effects by gases. Second, the suggested physical model is the official method used to characterize solar radiation by COMS Meteorological Data Processing System products (CMDPS) of KMA over the region [53]. The details of this physical model are as follows [18,51,54]:

(1) $$S_{T}=S_{I}+S_{R}+S_{A},$$

(2) $$S_{I}=S\left(\tau_{O}\tau_{R}-\alpha_{W}\right)\tau_{A},$$

(3) $$S_{R}={S\tau }_{O}\left(0.5\left(1-\tau_{R}\right)\right)\tau_{A},$$

(4) $$S_{A}=S\left(\tau_{O}\tau_{R}-\alpha_{W}\right)F_{C}\omega_{0}\left(1-\tau_{A}\right),$$

(5) $$S=I{\left(\frac{d_{M}}{d}\right)}^{2}\cos\theta ,$$

where *S_T_*, *S_I_*, *S_R_*, and *S_A_* are the total solar radiation, direct irradiance, diffuse irradiance due to Rayleigh scattering, and diffuse irradiance due to scattering by aerosols, respectively. Detailed nomenclatures of the parameters used for the satellite-based physical model are presented in Table 2.

In the case of the applied physical model, the cloud mask algorithm was executed to discriminate between cloudy and clear areas and, thus, determine the extent of cloud attenuation, since cloud coverage is the most important factor influencing the attenuation of solar radiation upon passing through the atmosphere. For cloud masking, the COMS MI visible and IR channels were used to determine whether the observed radiation was brighter or colder than that of natural bodies [55]. For pixels assigned to cloudy areas, cloud attenuation was determined using visible reflectance and the corresponding solar zenith angle, since higher cloud reflectance indicates lower cloud penetration, whereas high cloud optical thickness indicates high cloud attenuation [18]. A more detailed description of the physical model can be found in previous studies [52,54].

### 3.2. Aritificial Neural Networks (ANNs)

In this study, solar radiation on the surface of South Korea was simulated using COMS MI multispectral bands and a multilayer feed artificial neural network (ANN) operated employing the Levenberg–Marquardt back propagation (LM-BP) approach [56,57]. LM-BP, which is a second-order non-linear optimization technique, is not only usually faster and more reliable than other BP variants [58], but also provides a numerical solution to the estimation problem by minimizing the sum of the non-linear least square errors between the observed and predicted outputs in an iterative manner [59,60]. The number of hidden nodes should be carefully determined, since an overly low number of these nodes complicates network training, while an overly high number of nodes may result in overfitting. Therefore, trial-and-error testing was used to determine an appropriate number of hidden nodes. In addition, an early-stop training approach was implemented for generalization performance by splitting collocated data into three parts (training, validation, and test ones), since it is simple to understand and implement and is superior to regulation methods in many cases [61]. The MATLAB neural network toolbox was used to simulate the neural network LM-BP algorithm.

### 3.3. Regression Version of Support Vector Machine (SVM) 

Support vector regression (SVR) is a regression version of SVM that performs classification and, similarly to SVM, is well-suited for modeling small samples with powerful predictability [62,63]. Consequently, SVR/SVM has been used to estimate atmospheric parameters in many studies [63,64,65,66,67,68]. SVR transforms the original feature space into a high-dimensional one to find an optimal hyperplane, employing kernel functions such as Gaussian, radial basis, linear, and polynomial ones to effectively separate data [69,70]. Herein, SVR was implemented by MATLAB 2018a through the fitrsvm function [71] (http://mathworks.com/help/stats/fitsvm.html) containing the “KernalScale auto” module, which selects an appropriate scale factor using a heuristic procedure based on subsampling, and the “Standardize” module, which standardizes each variable using mean and standard deviations.

### 3.4. Random Forest (RF) 

The RF approach, which employs classification and regression trees (CART), comprises numerous decision trees (500 trees in this study) [72] and has been successfully used to predict various atmospheric variables [73,74,75,76,77]. Importantly, the RF method adopts two major randomization processes to overcome the limitations of CART (e.g., overfitting). These randomization processes select a random subset of training samples (at each tree) and variables (at each node) [63,78]. Independent trees (500 trees) are combined to predict an unknown pixel by averaging the results of trees. Consequently, the RF approach provides relative variable importance (i.e., the contribution of a given variable) based on the increase of the mean squared error as percentage (%IncMSE). In this study, RF analysis was conducted using the “Ranger” package (Version 0.10.1 with default setting) of R software, which implements RF analysis faster than the “Random forest” package. 

### 3.5. Deep Neural Networks (DNN) 

**Figure 2:**
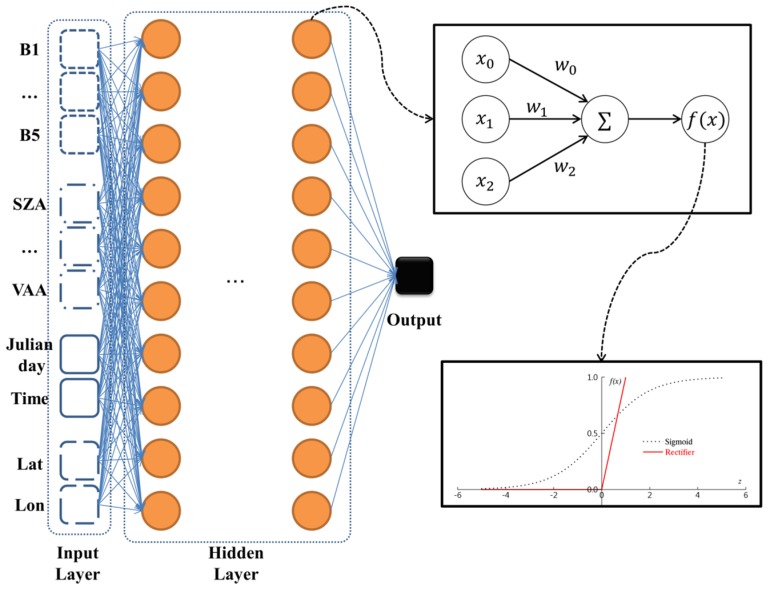
Structural diagram describing deep neural network (DNN) operation.

The DNN model is a machine learning method that has advanced based on ANN and is capable of trained complex input and learning procedures [79,80]. Herein, we used the H2O deep learning library of python for retrieving solar radiation based on COMS MI data. H2O is freely distributed software that can be easily exploited by various platforms such as Linux, Windows, and MacOS with excellent performance. A detailed description of H2O can be found at h2o-tutorial of github [81] and in the work of Arora et al. [82]. Deep learning is achieved by the use of a feed forward network and error back propagation to determine the weight of hidden nodes using a true variable (solar radiation observed by pyranometer). Like other deep neural network methods, the employed model comprised one input/output layer and multiple hidden layers (Figure 2). Various structures of the deep neural network (Table 3) were analyzed to determine an optimal training model to retrieve solar radiation. Prior to structural analysis, we set the “Rectifier” as an activation function of each hidden node that determined whether or not the input information reflected the nodes of next hidden layer. The above function has been widely used to train deep learning models because of its non-saturating non-linearity and fast convergence of the stochastic gradient descent [83,84]. Since the number of input variables equaled 14, the number of hidden nodes was chosen to be a multiple of this value. Although the corresponding relationship is not linear, we assumed that generally structure complexity is positively correlated with prediction accuracy. Each hidden node was tested according to three types of hidden layers, whereas the number of hidden layers increased with increasing number of hidden nodes. L1 and L2 regularizations were employed to avoid the over-fitting of training data. According to Cook [85], the L1 regularization reduces complexity by setting some close-to-zero weightings to zero, while the L2 regularization attempts to keep the overall weight close to zero. After analysis of the structure, the best DNN model selected 6 hidden layers including 140 hidden nodes for each hidden layer with 1 × 10^−5^ as the L1 regularization.

## 4. Results

### 4.1. Validation Using Data Supplied by Ground Pyranometers in South Korea 

One of the important objectives of training a machine learning model is the evaluation of the generalization performance of a new dataset. Moreover, it is also necessary to obtain reliable estimates of model generalization errors by objectively validating their spatial assessment performance using ground reference data. The methods used to validate data-driven and physical models can be broadly divided into holdout validation and k-fold cross-validation based on the separation of the total dataset for model training and testing, with each validation approach having inherent pros and cons [86]. This study mainly aimed to assess the performance of various methods from physical and data-driven models for retrieving the spatial distribution of solar radiation on the surface based on COMS MI satellite imagery. Therefore, the criteria that separating training and test datasets is based on locations of ground sites randomly for both validation approaches to evaluate the spatial assessment of solar radiation. In a hold-out method, 27 out of a total of 35 ground sites (75%) were used for training data (including 25% used for validation), and the remaining eight ground sites (25%) were used as a test dataset. The total volume of the match up data-set equaled 887,336, which implies that the hold-out approach can effectively evaluate model performance owing to the abundance of sample data.

**Figure 3:**
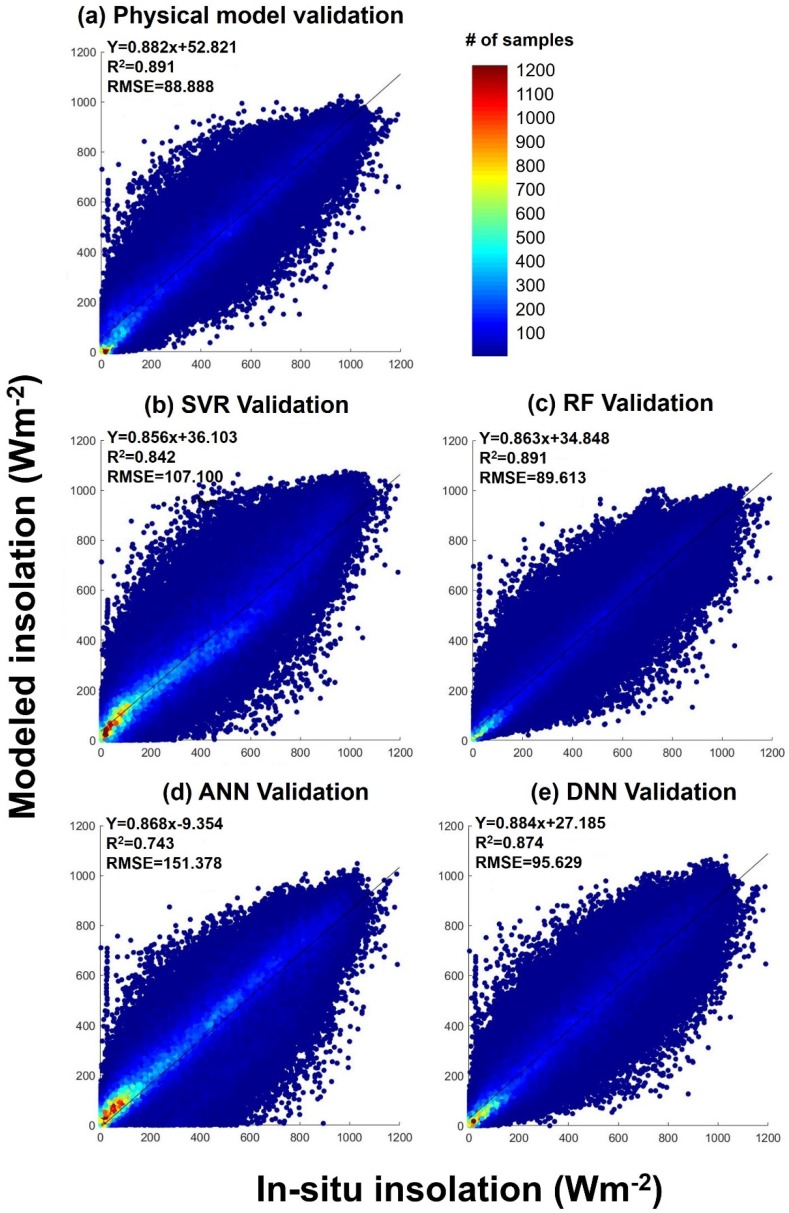
Density scatterplots describing the correlation between data provided by selected satellite imagery-based solar radiation retrieval models and the ground for (**a**) physical, (**b**) support vector regression (SVR), (**c**) random forest (RF), (**d**) artificial neural network (ANN), and (**e**) DNN.

Figure 3 shows density scatterplots obtained using the hold-out validation approach and describing the correlation between data provided by (i) selected satellite imagery-based solar radiation retrieval models and (ii) ground pyranometers located in South Korea (Figure 1). The estimated solar radiations obtained in both cases were compared with instantaneous ground solar radiation (in this case, with the results of hourly measurements) for all sky conditions (overcast, mixed, and clear). Figure 3 shows that all models featured patterns leaner than those of pyranometers, revealing that most match-up points are located around the centered one-to-one reference line. In addition, the density of instantaneous all-sky conditions appears to be higher in the area of low solar radiation, since it not only accounts for the effect of clouds, which is the most important attenuation factor of incoming solar radiation, but also for two cases of low solar elevation angle (sunrise and sunset) induced by the observation characteristics of the geostationary satellite.

Among the selected models, the physical one featured the highest statistical accuracy (root mean square error (RMSE) = 88.888 W·m^−2^, R^2^ = 0.891), although the second highest accuracy of the RF model (Figure 3c; RMSE = 89.613 W·m^−2^, R^2^ = 0.891) was not significantly different. Thus, it was concluded that the RF model should be able to simulate spatial solar radiation effectively and accurately using limited ground reference data, since the corresponding decision tree or SVM with kernels are believed to have two layers [87]. The third highest accuracy was obtained for the DNN model (RMSE = 95.629 W·m^−2^, R^2^ = 0.874). In this study, we expected DNN to provide the most meaningful results, since its deep and complicated neuron structures allow it to effectively simulate the spatial variation of solar radiation under the equally limited satellite data condition. However, this model was not the most accurate one among data-driven models. Nevertheless, the differences in accuracy between the previous physical model and the RF and DNN methods were well within the significant error range, i.e., machine learning methods used for the RF and DNN models with more than two network layers accurately estimated solar radiation modeling performance, even though optimized models from limited ground locations were employed. Conversely, the ANN (RMSE = 151.378 W·m^−2^, R^2^ = 0.743) and SVR (RMSE = 107.1 W·m^−2^, R^2^ = 0.842) models with shallow network structures featured remarkably lower accuracies than other selected methods.

**Figure 4:**
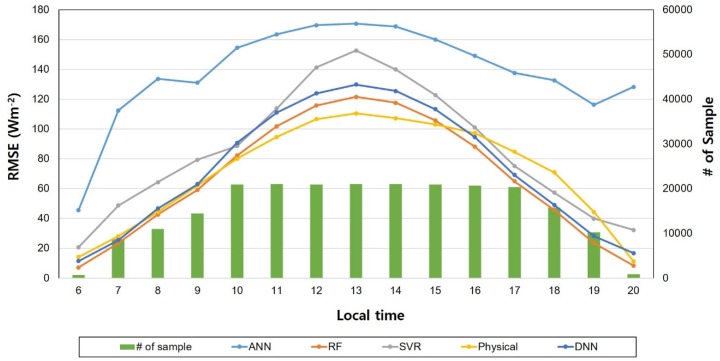
Temporal varations in root mean square error (RMSE) and sample number for each model by local time.

In addition, we evaluated the temporal variations of statistical results for each model to determine the dependencies on the number of data samples selected by the holdout approach, since the performance of data-driven models is mainly dependent on the number of samples used to train the network. For solar radiation in particular, the number of matchup datasets were mainly changed by solar location during daytime. Noon during the day with the highest solar elevation had relatively fewer samples while intermediate solar elevation cases could acquire relatively more data samples since the solar zenith angle (the most effective parameter in solar radiation) has the same value twice as the sun rises and falls (except noon) during the daytime. Figure 4 shows the variations of RMSE for the selected models and the corresponding sample number according to local time, clearly showing that the accuracy of the physical model was highest in spite of having the smallest sample number at noon during the daytime. In addition, the accuracy of the DNN and RF machine learning (ML) models was better than that of the physical model due to relatively better accuracy when the sun was rising and falling, meaning that the DNN and RF models effectively simulated solar radiation with reliable accuracy. Also, the ANN and SVR ML models showed lower accuracy, meaning that the applicability of these models to estimate solar radiation in the study area is limited due to their high sensitivity to sample number.

We also performed five-fold cross-validation to more objectively evaluate the spatial generalization capability of the suggested models. In k-fold cross-validation, the total dataset is randomly partitioned into k equal sub-parts, one of which is rotationally used as a test data set, while the remaining k − 1 sub-parts are used as a training data set. Although the validation processes increases with k, it is appropriate to evaluate how the result of a statistical analysis represents the generalization of spatial variation for data-driven models. Cross-validation was also used to test the data-driven model’s ability to simulate new areas of solar radiation that were not used for model training to flag the problems of overfitting or selection bias [88].

Table 4 shows the results of five-fold cross-validation for each selected model to interpret the generalization of solar radiation spatial assessment. As in the case of the holdout validation approach, the best results (RMSE = 87.441 W·m^−2^, R^2^ = 0.888) among data-driven models were obtained for the RF model. On the other hand, the DNN model, ranked as the third most accurate by holdout methods, was ranked second most accurate (RMSE = 88.219 W·m^−2^, R^2^ = 0.886). The physical model ranked most accurate by the hold-out approach was ranked as the third most accurate model (RMSE = 91.787 W·m^−2^, R^2^ = 0.882) in cross-validation (Table 4). However, the differences in accuracy between these methods were insignificant, as in the case of previously described holdout validation results. In addition, ANN featured the lowest accuracy among the selected models, similar to previous holdout validation results, while the SVR model featured the second lowest accuracy (Table 4). Thus, we inferred that increasing the complexity of a network structure is useful for simulating the spatial patterns of solar radiation using only time, observation geometry, and satellite spectral bands. According to the statistical analysis results, network structures with high complexity not only show robust simulation accuracy for the ground true site area but also exhibit spatial simulation capabilities for the untrained area that can be used to reliably estimate solar radiation by integrating satellite information. Therefore, one can improve spatial estimation characteristics using neural networks with a complicated and deep structure even when only simple satellite spectral bands are utilized without understanding the physical process of solar radiation.

In contrast, the physical model is considered to be less dependent on ground observation sites, since unlike machine learning methods, it does not use ground pyranometer data to fit algorithm models. However, even if the physical model is based on physical process parameterization [89], there still may be an accuracy deviation according to region location, as shown in Figure 3 and Table 4. In particular, since cloud coverage is the biggest error factor in the estimation of solar radiation and is subject to large regional variation [19,54], it is likely to result in spatial deviation of solar radiation.

### 4.2. Analysis of Variable Importance from RF 

**Figure 5:**
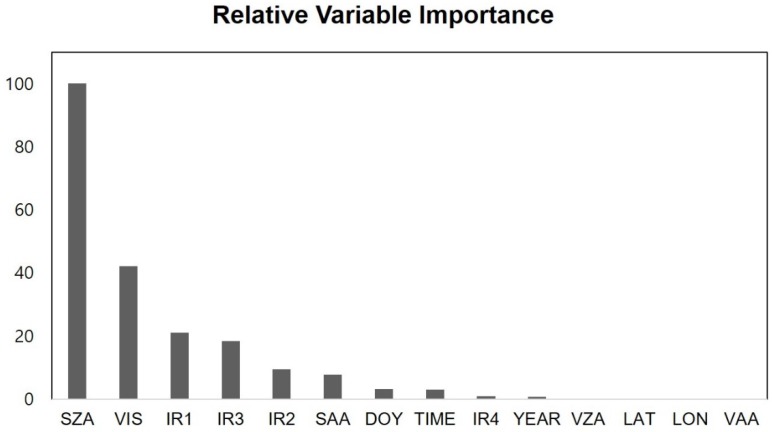
Relative variable importance determined using RF analysis for solar zenith angle (SZA), visible spectral band (VIS), infrared bands IR1–4, solar azimuth angle (SAA), day of year (DOY), time, year, viewing zenith angle (VZA), viewing azimuth angle (VAA), latitude (LAT), and longitude (LON).

RF analysis provides relative variable importance, which implies how the variable contributes to estimating solar radiation when mainly integrate with spectral bands from the COMS MI satellite. This is based on the difference between prediction error (mean squared error; MSE) from original variables and after permuting each variable. Many remote sensing studies have used variable importance in this way [32,48,72,77,79,90,91]. Figure 5 shows the relative importance of input variables for estimating solar radiation. As expected, solar zenith angle and visible spectral band showed the highest variable importance when estimating solar radiation from ML models. The former directly determines the amount of solar radiation related to solar elevation and has a high sensitivity to cloud radiation attenuation [57] while the latter is linearly related to cloud penetration, so these have a high relative importance (Figure 4). In addition, the contributions of the IR1 and IR3 satellite bands were higher than IR2 and IR4. As each IR band is determined by wavelength, IR1 is more important for estimating solar radiation than other IR bands since it is effectively estimating the brightness temperature of cloud tops using the thermal characteristic of the wavelength (10.8 μm). In contrast, the geographically fixed variables had narrow variability over South Korea, so latitude, longitude, viewing zenith angle, and viewing azimuth angle were less important for estimating solar radiation (Figure 4). Contrary to our expectations, the contributions of latitude, longitude, day of year, and time were low; we considered these variables to have multicollinearity to solar zenith angle and solar azimuth angle (SAA). 

## 5. Discussion

We also produced solar radiation maps of the region using each of the selected methods to evaluate their performance visually for predicting the spatial distribution of solar radiation. We calibrated the ML models and produced the maps using 9 of the 14 variables, selected by importance analysis (Figure 5); the eliminated variables were location-sensitive and did not allow station-based datasets to be applied to an expanded area. The year variable had little variation when compared to that of solar radiation; furthermore, solar radiation changes with time were well reflected in UTC and Julian day.

**Figure 6:**
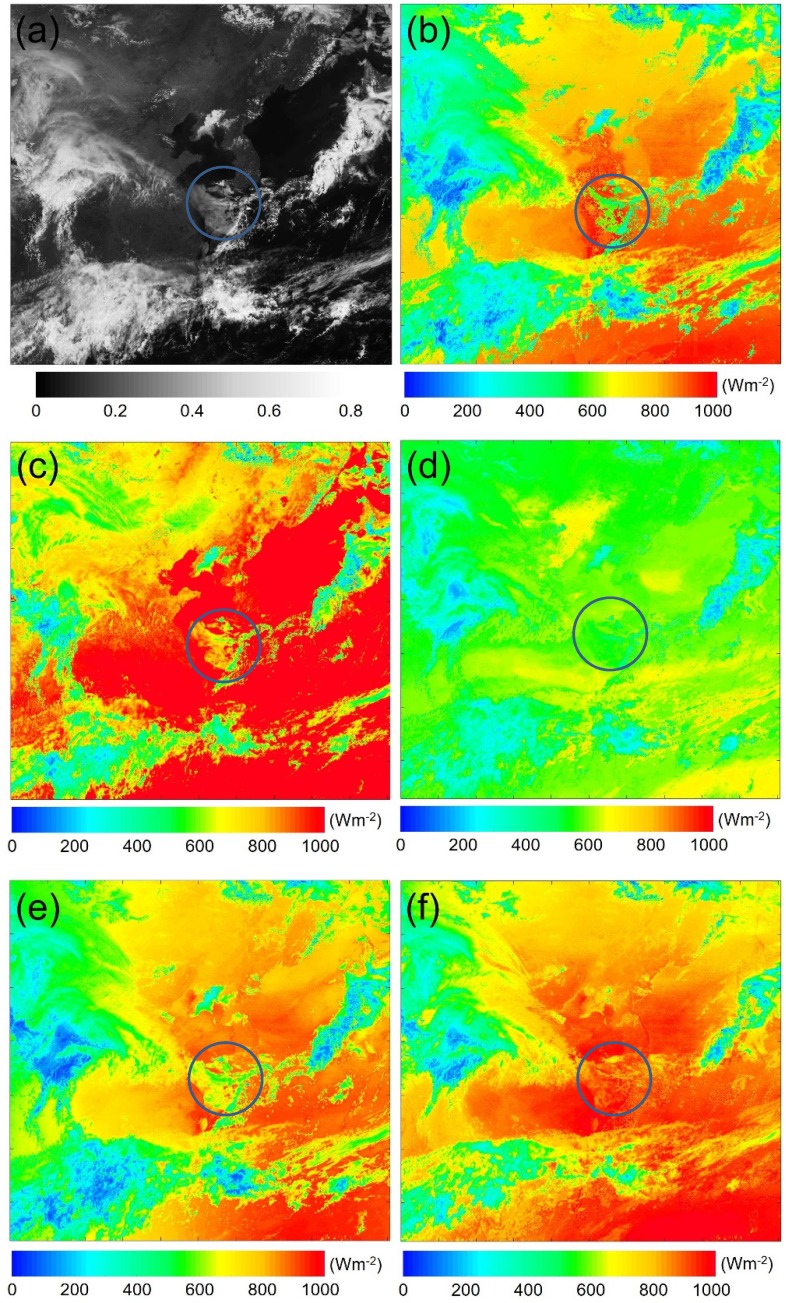
Comparison of solar radiation maps simulated using (**a**) the pattern determined from the visible band on 03:00 UTC, 15 April 2017 with (**b**) the physical model, (**c**) SVR, (**d**) RF, (**e**) ANN, and (**f**) DNN.

In Figure 6a, the visible band imagery of COMS MI acquired at 03:00 UTC April 15, 2017 was used as comparative reference imagery, since it accurately described the spatial pattern of clouds over the region. In other words, it should be possible to evaluate the performance of a given model indirectly for the spatial assessment of solar radiation by interpreting how well this model estimates the spatial pattern of clouds, since cloud coverage is the most important parameter influencing solar radiation on the surface [18,19] and one of the most spatially variable atmospheric constituents [92]. However, it is difficult to evaluate quantitative values with visual inspection of visible band image, indicating that ground measurements for a much larger domain should be of use for assessment of the their intensities. 

For solar radiation estimation by the physical model (Figure 6b), the cloud factor comprising the contributions of cloud reflectance and solar zenith angle was applied according to the presence of clouds to reflect radiation attenuation. Therefore, the results of cloud masking directly affected the spatial value of solar radiation. The spatial pattern of solar radiation attenuation due to clouds was similarly compared to those obtained using visible channels (Figure 6a), where the cloud pattern was accurately described. The high reflectance of clouds (shown as a white area) means that thick clouds corresponded to low solar radiation values, while transparent thin clouds showed moderate values of solar radiation compared to clear-sky areas. However, when viewed from the blue circle in Figure 6b, the thin cloud area in the visible image in Figure 6a was classified as a clear-sky area in the physical model. Therefore, the value of solar radiation was overestimated despite the presence of thin clouds, since it is not easy to classify cloud areas (especially thin transparent clouds) using limited satellite spectral bands [75].

The solar radiation maps estimated by SVR, RF, ANN, and DNN (Figure 6c–f) revealed the spatially distinctive intensities of solar radiation when compared with the physical model. The SVR-estimated solar radiation featured a wider range than that of the physical model (Figure 6b,c), making it hard to identify the overall spatial pattern of solar radiation because this was optimized for biased values. Notably, SVR accurately estimated the spatial pattern of solar radiation over thin clouds that was overestimated by the physical model in the blue circle in Figure 6b. On the other hand, the RF result (Figure 6d) produced a narrow range of solar radiation concentrated around 500 W·m^−2^. Although both hold-out validation and five-fold cross-validation showed good performance (Figure 3 and Table 4), the spatial distribution of solar radiation was rather underestimated. ANN accurately predicted the spatial cloud pattern in comparison with the visible image and physical model (Figure 6e), but the poor results obtained for both hold-out and five-fold cross validation methods made it difficult to simulate a reliable solar radiation map. In other words, although the spatial characteristics could be accurately simulated, it was difficult to estimate solar radiation precisely with limited data due to ANN’s shallow network structure. DNN accurately simulated complicated cloud spatial distributions (Figure 6f), but also specifically described the attenuated intensity patterns due to cloud coverage despite using only time, observation geometry, and five spectral bands of COMS MI. Notably, DNN also accurately simulated the spatial distribution of thin clouds in the blue circle, whereas these areas were overestimated by the physical model because of the cloud masking error. However, the overall solar radiation of DNN model was overestimated compared with the physical model. 

What we should be aware of is that the physical model also has an error in the aforementioned statistical analysis. There were clear limitations to determining the generalized spatial assessment using only visual inspection of a single image. Although we used a large quantity of pyranometer data, all locations were restricted to South Korea, so one main reason for the different solar radiation intensities of the data-driven model related to the limited ground-measurement domain used to train the models. This made it difficult to generalize results for the relatively small South Korean region to the larger northeast Asia region. Unfortunately, it was also difficult to obtain well-calibrated ground-measurement data with hourly temporal resolution from other nearby countries.

Nevertheless, the data-driven model integrating satellite spectral bands showed the most potential for simulating spatial patterns especially for clouds over Northeast Asia region. Future studies should work toward spatial assessments of solar radiation using wider coverage of ground reference data.

We used a physical model modified from Kawamura et al. [51] among numerous independent models [28] as a comparative model to evaluate the generalization capability of data-driven models. The satellite-based Kawamura physical model was used mainly because it is employed in the official solar radiation products of CMDPS from KMA [53], i.e., its algorithm is continuously and officially optimized by checking its accuracy by comparison with ground measurements and relevant input data. In addition, we placed high priority on the consecution of solar radiation when selecting the physical model over the study area. Various geostationary satellites such as Geostationary Meteorological Satellite-5 (GMS-5), Multifunctional Transport Satellite-1R (MTSAT-1R), and COMS MI have been continuously operated in the Northeast Asia area since the 1990’s, and algorithms for solar insolation prediction obtained for geostationary satellites GMS-5 [51], MTSAT-1R [18], and COMS MI [54] were based on the Kawamura physical model and optimized according to the characteristics of payload sensors.

## 6. Conclusions

This study used four ML approaches to estimate solar radiation. According to the statistical analysis results, the predictions of the selected models were in good agreement with data supplied by ground pyranometers in South Korea. In both hold-out and cross-validation approaches, the physical model, RF, and DNN showed similarly good performance within the significant error range. Specifically, according to the five-fold cross-validation method, the physical, RF, and DNN models featured R^2^ values of 0.882, 0.888, and 0.886, respectively, meaning that deep and complicated neuron structures allowed effective simulation of the spatial variation of solar radiation despite limited satellite data. When compared to previous studies, statistical values of the prescribed methods were reasonable [93,94], while the R^2^ values of ANN and SVR were 0.791 and 0.836, respectively. We inferred that the shallow network structures of SVR and ANN made it difficult to interpret the relationship between satellite spectral data and incoming solar radiation on the surface for a limited ground area, since linear SVR and logistic regression can be attributed to a single-layer processor. In addition, we analyzed the hourly RMSE variations of each model and showed that the temporally dependent accuracies of ML models were mainly due to differences in sample number. RF and DNN showed relatively less sensitivity to sample number while SVR and ANN had more dominant time-dependent variations in accuracy.

Lastly, although RF and DNN showed good modeling performance for ground measurements, the spatial distribution of solar radiation predicted by these models was underestimated or overestimated when compared with the physical model. For RF, the spatial values of solar radiation were concentrated around the mean (~500 W·m^−2^) because this model was developed based on the lowest prediction error [75]. Many studies have been conducted to overcome these problems by oversampling or using cumulative density function matching [90,91]. In comparison, DNN showed the second-best results in modeling performance but overestimated its spatial distribution when compared with the physical model. The main reason for this poor performance originated from bias introduced by limited ground references and non-negligible sample-dependent accuracy of the model. Nevertheless, although the solar radiation maps of the data-driven model simulated different intensities due to the absence of ground references covering more of the region, it accurately simulated complex spatial patterns of clouds. This indicated that the combination of ML methods and satellite spectral imagery has the potential to produce useful spatial radiation maps. 

In conclusion, we found it particularly encouraging that RF and DNN, which have more than three layers of networks, could accurately simulate the challenging spatial pattern of thin clouds, whereas the physical model failed to do so because of cloud mask error. One of the largest difficulties faced by the physical model is that it attempts to classify thin cloud areas precisely based on limited satellite data, and the misclassified results eventually affect the final distribution of solar radiation. However, RF and DNN overcome these limitations of the physical model by using limited satellite data only, and the results obtained are therefore expected to increase the utilization of the RF and/or DNN approach for solar radiation field estimation in the future.

## Figures and Tables

**Table 1:** Detailed characteristics of Communication, Ocean, and Meteorological Satellite (COMS) Meteorological Imager (MI) used to estimate solar radiation in the study area.

Satellite Sensor	Orbit Type (altitude)	Wavelength (μm)	Spatial Resolution	Application
COMS MI	Geo-synchronous (36,000 km)	VIS: 0.55–0.80	1 km	Cloud detection in daytime, atmospheric motion vector
		IR3: 3.50–4.00	4 km	surface temperature
		IR4: 6.50–7.00		Assessment of water vapor
		IR1: 10.30–11.30		Cloud detection using IR split window method
		IR2: 11.50–12.50		Cloud detection using IR split window method

**Table 2:** Description of parameters used for estimation of satellite-based solar radiation.

	Parameter
c	Sun-earth distance
$d_{M}$	Sun-earth distance (annual mean)
$F_{C}$	Ratio of forward to total scattering by aerosols
I	Solar constant
S	Incident solar constant
$S_{D}$	Diffuse irradiance
$\alpha_{w}$	Absorption of water vapor
θ	Solar zenith angle
∅	Solar azimuth angle
$\tau_{A}$	Transmittance due to attenuation by aerosols
$\tau_{O}$	Transmittance due to absorption by ozone
$\tau_{R}$	Transmittance due to Rayleigh scattering
$\omega_{O}$	Single scattering albedo

**Table 3:** Parameters of the training model used to find an optimal structure for retrieving solar radiation.

Structure	Configuration			
Number of hidden nodes	14	70	140	210
Number of hidden layers	4–6	5–7	6–8	6–8
L1 regularization	0, 1 × 10^−4^, 1 × 10^−5^			
L2 regularization	0, 1 × 10^−4^, 1 × 10^−5^			

**Table 4:** Statistical performance of selected models determined by five-fold cross-validation.

Methods	R^2^	RMSE ^1^ (W·m^−2^)	MAE ^2^ (W·m^−2^)	Slope
Physical model	0.882	91.787	66.247	1.041
SVR	0.836	106.185	76.964	0.862
RF	0.888	87.441	60.603	1.014
ANN	0.791	123.211	91.240	0.912
DNN	0.886	88.219	60.817	0.901

^1^ RMSE: Root Mean Square Error, ^2^ MAE: Mean Absolute Error.
